# 

**Published:** 1860-05

**Authors:** 


### SUBJECTS FOR DISCUSSION,

*By the Western Dental Society, at its next meeting, to be held in Springfield, Ill., on the fifth day of July,* 1860.

FIRST. What are the effects upon the permanent teeth, of ulceration about the roots of the deciduous teeth?

SECOND. Causes of caries of the teeth, remote and immediate.

THIRD. What are the indications to be met in, and the end to be attained by the filling of a root? What is the best method of performing the operation? What is the physiological or pathological condition of a filled root? and what is the average of success in this branch of practice?

FOURTH. Method of extracting the several classes of teeth and roots.

FIFTH. Etiquette, embracing fraternal intercourse, (beginning with, who is my brother?) Treatment of neighboring practitioners. Demeanor to patrons. Courtesy to physicians. Courtesy to clergymen, etc., etc.

SIXTH. Mechanical Dentistry—Gold plates, continuous gum, hard rubber, etc., etc. What is their comparative value for dental uses, and what should decide our choice between them in any given case?

A. W. FRENCH,<br>E. A. BOGUE.<br>C. A. FITCH, } *Committee.*

### AMERICAN DENTAL CONVENTION.

The Executive Committee report the following *Order of Business* for the meeting to be held at Saratoga, on Tuesday the 7th of August, 1860.

1st. Reading Minutes of last meeting.
2nd. Reports of Officers and Committees.
3rd. Admission of Members.
4th. Election of Officers.
5th. Retiring President's address.
6th. Induction of Officers elect.
7th. Miscellaneous Business.
8th. Essays and Discussions.

All Essays shall be read to open the discussions on the subjects to which they relate and they shall be limited to 20 minutes in length.

I. Artificial Dentures.
Preparing the Mouth.
Materials for and modes of obtaining impressions and models.
Various Basis and their Manipulations.
*Metals.*
*Minerals.*
*Gums.*

II. Structure and Nutrition of the Teeth.

III. Irregularities. *Causes.*
*Treatment.*—Prophylactic. and Remedial.

IV. Sensitive Dentine. *Pathology.*
*Cause,*
*Treatment.*

V. Exposed or Wounded Pulp.
*Prognosis.*
*Treatment,*—Preservative, and when devitalized.

VI. Diseased Dentine.
*Pathology.*
*Causes,*—Remote and proximal, including the effect of different diseases and medicaments upon the teeth.

*Treatment*,—Prophylactic, *general and local.*
Remedial, *general and local* including materials for and modes of filling.

N. B.—No member to speak more than fifteen minutes or more than twice upon the same subject without permission.

9th. Exhibitions of Models, Improvements or Inventions with Miscellaneous Discussions or unfinished business.

GEO. F. FOOTE,

JOSEPH RICHARDSON,

T. H. BURRAS,

JEREMIAH MASON,

S. W. ROBINSON.

} Executive Committee.

### THIRTEENTH VOLUME

OF THE

### DENTAL REGISTER OF THE WEST,

EDITED BY

**J. TAFT and GEO. WATT.**

**Issued Monthly. - - - $3 a year in Advance.**

The Volume commences in July. CONTRIBUTIONS to the pages of the Register respectfully solicited.

Exchanges and Correspondents will please address "Dental Register," or J. TAFT, Cincinnati, O; Business communications to JNO. T. TOLAND.

The Register being issued Monthly is always fresh, and in advance of all others, therefore indispensable to the practitioner who desires to keep posted up with the new inventions and improvements which are daily developed.

Neither expense nor effort will be spared to give value and variety to its contents. Articles will be illustrated with well executed engravings, whenever they will add to the interest or assist in explanation.

Its extensive circulation and frequency of issue makes it the BEST DENTAL ADVERTISING MEDIUM in the United States.

### TERMS OF ADVERTISING.

| | One Year. | 6 Mo. | 3 Mo. | 1 Insertion. |
|---|---|---|---|---|
| One Page, | $50 | $30 | $20 | $15 |
| Half | 30 | 20 | 15 | 10 |
| Quarter | 20 | 15 | 10 | 7 |
| One-Eighth | 15 | 10 | 7 | 5 |

**JNO. T. TOLAND, Pub. and Proprietor,**

38 *West Fourth Street, Cincinnati, Ohio.*

Subjects for Discussion by the Western Dental Society, at its next Meeting,.... 611

